# Time-Motion Analysis of Patient Flow in a Family Medicine Department of a Tertiary Care Hospital in Lahore

**DOI:** 10.12669/pjms.42.(ICON26).15710

**Published:** 2026-04

**Authors:** Humayon Khalid Saeed, Osama Tahir, Aqsa Mairaj

**Affiliations:** 1 Humayon Khalid Saeed, MBBS, MBA, MCPS, MRCP1. Head of Clinical Operations, Indus Hospital and Health Network, Lahore, Pakistan; 2 Osama Tahir, BS Software Engineering. Deputy Manager – Applications, IT Department, Indus Hospital and Health Network, Lahore, Pakistan; 3 Aqsa Mairaj Assistant Manager Clinical Operations, Indus Hospital and Health Network, Lahore, Pakistan

**Keywords:** Time-Motion, Patient Journey, Family Medicine, Tertiary Care Hospital, Workflow Analysis

## Abstract

**Objective:**

This Time-Motion study used a structured approach to analyze patient flow through the Family Medicine Department, to improve efficiency in healthcare delivery by identifying bottlenecks and opportunities for improving workflow.

**Methodology:**

This was a cross-sectional time-motion study and data for six-day-period between 08:00 A.M. to 05:00 P.M. was obtained from the Hospital Management Information System (HMIS) of Indus Hospital and Health Network, Lahore, that automatically records time stamps for key patient flow events (registration, consultation, investigation, and discharge) as part of routine clinical operations. Electronic time stamps were generated from HMIS. 2,762 patient journeys were included, each representing one complete patient visit to the Family Medicine Department during study period.

**Result:**

It was found that the average time in doctor’s chamber was approximately eight minutes (reasonable for quality consultation), yet subsequent processes like, recording vital signs, phlebotomy, ultrasonography etc, generated significant delays. Doctor-to-ultrasound pathway showed highest average time of 167.5 minutes. This study revealed that non-clinical and post-consultation processes consume a disproportionate share of patient time, overshadowing direct clinical encounters. Bottlenecks were linked to infrastructure and process design constraints.

**Conclusion:**

The study identifies significant time delays in diagnostic and pharmacy processes. The findings suggest system-level reforms particularly in increasing diagnostic capacities to optimize workflow.

## INTRODUCTION

A time and motion study serves as an effective method for evaluating the efficacy of various processes within the healthcare setting. The origins of time and motion studies date back to the early 20th century. Taylor^1^ introduced *time study* as part of his principles of scientific management, focusing on the measurement and standardization of task duration. In the same era, Frank and Lillian Gilbreth emphasized *motion study*, aimed at simplifying and improving work methods to enhance efficiency.^2^ Time and motion studies have evolved to integrate the two efficient techniques, laying the foundation for the modern ‘time and motion’ study framework, which has since been widely applied to evaluate workflow efficiency in healthcare and other industries.^3^

This framework serves to identify bottlenecks, inefficiencies and opportunities for optimization of tasks. This is achieved through the process of analyzing the various tasks and movements occurring within a healthcare setting, as well as the time spent on different activities by the respective healthcare providers, administrators and support staff. The synthesis of time-motion techniques has paved way for ‘lean’ healthcare evaluations, shifting focus from mere standardization to elimination of non-value-added activities that contribute to provider fatigue and reduce patient satisfaction.^4^,^5^

The importance of time-motion analysis has been a particular area of interest in recent studies, as an attempt to better understand the workload and productivity of healthcare professionals. Time-motion studies have further led to the identification of workflow optimization opportunities in individual departments.

Studies evaluating Outpatient Departments in tertiary care hospitals have reported various findings across South-Asia, with majority patients reporting dissatisfaction with time management.^6^ A cross-sectional observational study at the fever clinic of Midnapore Medical College and Hospital in India revealed majority of participants reported dissatisfaction with time management and only 18% expressed complete satisfaction.^6^ A study at the AYUSH hospitals showed that from first arriving at the hospital to the final exit, a patient was spending approximately two hours in the OPD and three minutes during doctor consultations, 16 minutes at the pharmacy. Physicians were dedicating majority of their time to documentation and administrative tasks. Patients were found to be least satisfied by OPD waiting time, consultation time, and pharmacy services.^7^,^8^ Likewise, physicians in outpatient settings are also found to devote a substantial portion of their time to non-clinical tasks, such as paperwork and communication, further limiting direct patient interaction.^8^,^9^

Recent localized research in Lahore evaluated that while clinical competency is high, patient satisfaction remains significantly tied to the efficiency of service delivery.^5^ Although some private tertiary settings in Lahore report high satisfaction with physician communication, there is a documented need to further investigate how operational bottlenecks specifically impact patient perceptions in various OPD environments.

The most significant source of time ‘wastage’ in Pakistani tertiary hospitals is reportedly the non-linear patient movement (patients being sent back and forth between counters).^10^ Data from EHRs can be used in predictive analysis through machine learning to predict ‘peak hours’, which is shown to reduce waiting time by 30-40%.^10^ Simultaneously, the transition to Electronic Health Records (EHRs) has potential to create a trust-tension paradox, digital fatigue and ‘dual burden’ effect, where digital documentation initially increases administrative time despite reducing physical motion. The “learning curve” for staff in Pakistan initially increases the time spent on documentation by 25% compared to paper records.^11^ While modern algorithms offer the potential to reduce physical bottlenecks in Pakistani tertiary care by up to 40%,^10^ the transition to digital record-keeping has introduced new administrative time burdens that can initially hinder clinical efficiency.^11^-^14^

Time-motion inefficiencies are not limited to physicians. Overall, the literature demonstrates that time-motion studies consistently reveal imbalances in task distribution.

There is an apparent lack of comprehensive research specifically focusing on the Family Medicine Department/ OPD within the tertiary care hospitals in Pakistan. Therefore, this study aimed to examine the intricate time-motion patterns in the Family Medicine OPD of a tertiary care hospital in Lahore, in order to identify gaps in patient services and opportunities for improvement. Specifically, it seeks to analyze the patterns affecting efficiency, convenience, and patient satisfaction, thereby contributing to building a more sustainable and resilient healthcare system for the future. The recent technological advancements within healthcare settings, such as the use of Electronic Health Records (EHRs) can further be explored in order to understand the impact of incorporating such technology on workflow efficiency by reducing time spent on documentation. According to the Pakistan Economic Survey 2024-25, despite an increase in health infrastructure, the rising patient-to-provider ratio in public tertiary care continues to strain service delivery, necessitating a strategic shift toward data-driven hospital management and operational efficiency.^15^

**Graph.1:**
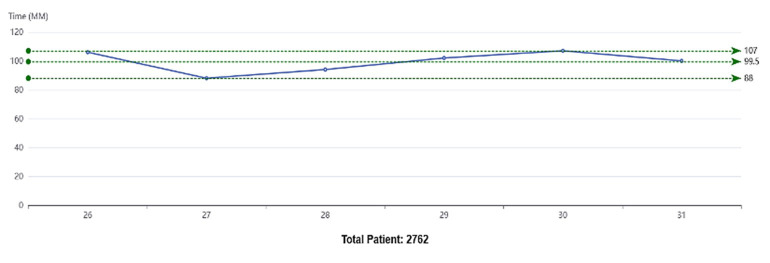
Total patient journey across six days showing variations in average waiting time and service durations. Note: X-axis represents observation days.

### Research Question:

How do the time and motion patterns of a patient’s journey in the Family Medicine Department of a tertiary care hospital in Lahore affect patient care efficiency, convenience and satisfaction?

### Objectives:

1. To conduct a time-motion study in the family medicine/OPD department of a tertiary care hospital in Lahore.
2. To identify gaps in patient service and explore opportunities for improvement.

## METHODOLOGY

This was a cross-sectional time-motion study. Data was collected through Hospital Management Information System (HMIS) records, and validated through direct observation, tracking patient journeys across 10 pre-defined service checkpoints in the Family Medicine Department Each checkpoint was time-stamped using the HMIS, which automatically records event times (e.g. registration, triage) synchronized with the central server clock. The duration at each checkpoint and transition interval was calculated as the difference between consecutive timestamps. Patient dropouts were noted when a process step was not completed. Average time at each service point, transitions, and dropouts were recorded to identify workflow bottlenecks. Data were compiled and analyzed descriptively using Microsoft Excel 2016 with averages, percentages and graphical summaries.

### Study Setting:

This study was conducted in Family Medicine Outpatient Department of a tertiary care hospital in Lahore over a six-day period between 08:00 A.M. to 05:00 P.M. The hospital caters to a low-to-middle income population seeking affordable, comprehensive care. The Family Medicine Department serves as a high-volume primary contact point receiving an average of 300 patients daily. Each patient interacts with multiple service points, creating potential bottlenecks in patient flow. This department represents these dynamics in LMIC context, providing clinically significant insights into workflow design, highlighting diagnostic and pharmacy inefficiencies.

### Sampling Strategy:

A total of 2,762 patients visiting the department during this period were included in the study. Each patient journey corresponded to a single patient’s visit, each patient that completed one full visit during the study period was included in the sample.

### Inclusion & Exclusion Criteria:

All patients attending the Family Medicine Department during the study period were included. Patients leaving the hospital before completing their patient journey were excluded.

### Ethical Approval:

This study was reviewed by the Institutional Review Board and it was determined that, this study is exempt from IRB review, as the use of human subjects, including their data, remain within the scope of the exemption category. IRB certificate number IHHN_IRB_2025_08_032, dated: September 10, 2025 was allotted.

**Graph.2:**
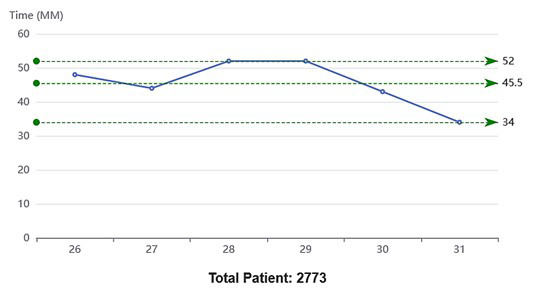
Average time spent in Doctor to Pharmacy segment of patient journey over a period of six days Note: X-axis represents observation days.

**Graph.3:**
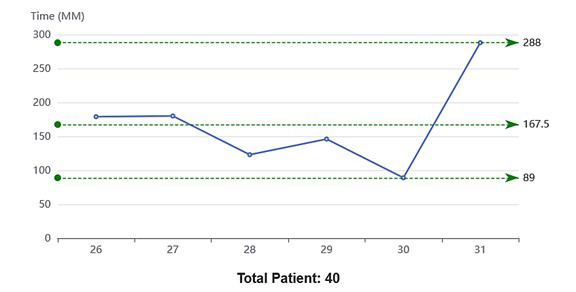
Average time spent in Doctor to Ultrasound segment of patient journey Note: X-axis represents observation days.

## RESULTS

The patients coming to OPD1/ Family wing falls in two groups; The ones who are coming to hospital for the very first time and will need New Registration having to pass through process of “Welfare”, and those that are already registered. In process of welfare, patients are evaluated for a group that will be treated from Zakat funds while the other will be treated from Sadaqaat funds. The patients who are already registered and have passed through welfare stage will be entered in HMIS with title of visit to hospital. However, each patient from both groups is entered for “Visit” for the same day. So, the total number of patients moving to Vitals include patients from both groups.

The Table-I shows that during the six-day period of the study, total of 1413 patients got Registered as new patients while 1387 patients moved to Welfare process. Twenty six patients discontinued journeys without progressing to further stages while 3264 patients who had their visits entered in HMIS i.e., both newly registered and old patients for follow ups, went to the vitals stage while 3264 patients moved to doctor chambers for doctor’s examination and advice. After doctor chamber visit, according to the diagnosis of disease, the patient journey splits into many directions i.e., some going to diagnostics tests like Laboratory tests, X-Rays, Ultrasounds and ultimately to pharmacy. Some patients who are not prescribed any tests, move to pharmacy to get their medicines while the other groups after getting their tests done finally move to pharmacy to get their medicines.

**Table-I:** Total patient flow and time distribution across each service checkpoint of the outpatient journey in the Family Medicine Department.

Station	Number of Patients	Minimum Time (Mins)	Maximum Time (Mins)	Average Time (Mins)	Standard Activity Time (Mins)	Waiting Time (Avg. – Activity Time)
Total Patient Journey	2762	88	107	99.5		
Registration to Welfare	1413	22	30	24.83	Reg.- 3, Welfare -5	16.83
Welfare to Vital	1387	6	12	8.83	Vitals – 3	5.83
Visit to Vitals	3264	19	25	21.17	Visit – 2	19.17
Vital to Doctor	3264	24	47	34.83	8	26.83
Doctor to Pharmacy	2773	34	52	45.5	3	42.5
Doctor to Lab	820	33	46	38.67	5	33.67
Doctor to X-Ray	132	20	41	30	7	23
Doctor to Ultrasound	40	89	288	167.5	15	152.5

The data shows out of 3264 patients seen by doctor, 820 (25.1%) cases went to laboratory tests, 132 (4.04%) were advised X-ray’s, while 40 (1.2%) were advised Ultrasound. Out of 3264 patients, 2772 (84.9%) patients went to pharmacy to get their medicines. During the six-day period, 491 patients did not get medicines as they were not prescribed medicines at this stage, or preferred to drop out during this stage or the medicines prescribed to them were not available in the pharmacy.

The maximum average time (167.5 min) was taken in Doctor to Ultrasound segment, significant average time of (45.5 min) was taken in doctor to pharmacy and average time of (38.67 min) for doctor to Laboratory segment.

To know about the actual waiting times in each segment, the researcher personally visited all stations to observe the time taken at each activity for multiple patients. The average times noted for each activity are mentioned in Table-I.

While comparing this average time of actual activity, the doctor to ultrasound time (152.5) was still the highest, followed by pharmacy having (42.5 min) as average time and doctor to lab average time of (33.67 Min). The vital to doctor time included the time in doctor chamber and average time of (26.83).

**Fig.1:**
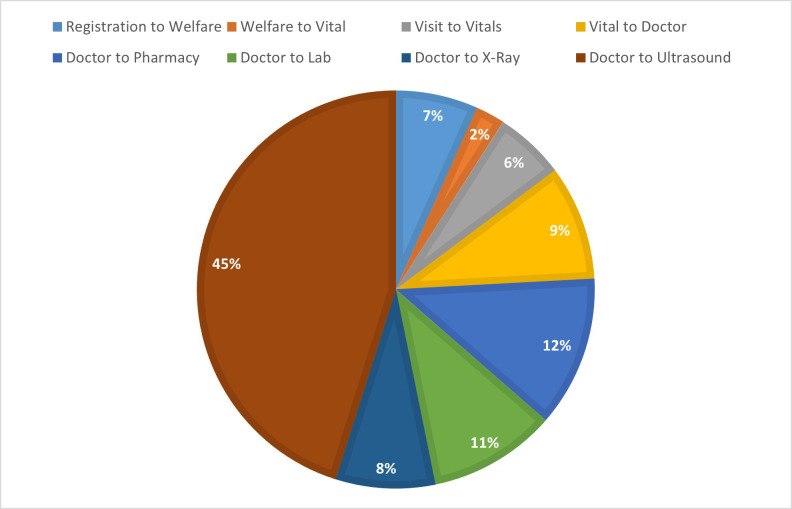
Percentage of total visit time spent at each service station in the patient journey.

The Fig.1 shows percentage of total time spent on each station. The highest amount of time was spent on Ultrasound i.e. 45% of total time. A significant portion of total time was spent in the Pharmacy, i.e. 12%, and the Lab, i.e. 11% of total time. The lowest amount of time was seen in Welfare to Vitals station, i.e. 2%.

## DISCUSSION

The findings from the current study highlight that while the average time spent in the physician consultation was reasonable, the greatest inefficiencies occurred in the diagnostic and pharmacy segments of care. The longest delays were observed between the physician consultation and ultrasound, with an average waiting time of 152.5 minutes, largely attributable to limited equipment capacity, despite adequate human resources. The doctor-to-ultrasound interval remained the longest, primarily due to limited machine availability despite adequate staff. The department with two machines performs ultrasound scans for patients from OPD1/FM, Consultant clinics, Indoor patients and also patients from emergency. Pharmacy waiting times were reported to be disproportionately high (42.5 minutes), primarily due to limited dispensing windows and staff, resulting in long queues. This was linked to only two dispensing windows managed by four staff members. These findings align with lean healthcare principles, emphasizing that efficiency is gained not just by working faster, but eliminating non-value-adding activities.^4^,^5^ The doctor-to-lab average time (33.67 Min) revealed that staff of two members was working as team where one member was entering data in system while the other drew samples as phlebotomist and collected other samples like urine and stools.

Comparable limitations with inadequate pharmacy staffing and infrastructure in low-and-middle income countries results in patient dissatisfaction.^5^-^7^,^16^,^17^ While laboratory services demonstrated more efficient output, delays still existed, reflecting the burden placed on small teams tasked with balancing both administrative and technical tasks, resulting in service delays.^8^,^9^,^12^,^13^ Recent evidence suggests machine-learning algorithms can predict peak hours and optimize patient flow by anticipating surges in demand and allocating machine times accordingly.^10^,^11^

The vital to doctor time included the time in doctor chamber and average time of (26.83). Average consultation time in doctor chamber was around eight minutes, which is reasonable for maintaining care quality. Overall, our results mirror evidence from similar studies in South Asia, where patients often spend several hours in outpatient departments, with only a fraction of this time devoted to direct clinical interaction.^5^-^8^ These inefficiencies underline the need for system-level interventions, including expansion of diagnostic capacity, redesign of pharmacy workflows, and task-shifting of non-clinical duties.

The present study has brought to the surface certain gray areas requiring urgent attention for sustainable care. Patient satisfaction is widely recognized as a core indicator of service quality and overall system’s performance at any level of health services.^5^,^10^,^19^ Contributors include queueing delays, limited facilities and poor signage. Beyond equipment and staffing shortages, contextual factors such as queuing systems, waiting area facilities, and signage contributed to inefficiencies. Poorly designed outpatient environments can amplify delays and negatively influence patient perceptions of care quality.^18^-^20^ These factors all act as hidden contributors to inefficiency, requiring a systems-level approach to address them. Simultaneously, an emphasis is needed on workflow redesign, investment in diagnostic capacity, and use of electronic health information technologies to streamline patient flow. It is also crucial to remain prepared for the ’dual-burden’ effect observed in other Pakistani institutions.^11^-^14^ To avoid this, our suggested workflow redesign must include adequate training and support systems to ensure that Electronic Health Records (EHRs) act as an efficiency tool rather than an administrative hurdle that detracts from physician-patient interaction time.

### Strengths of the Study:

The study combined direct observations of the researcher with HMIS records, allowing objective documentation of patient flow.

### Limitations of the Study:

The study was limited to a single department and short duration, which may not capture variability across time or different hospital units.

## CONCLUSION

This study revealed that while physicians’ consultation times were generally appropriate, significant inefficiencies existed within diagnostic and pharmacy services. These were observed due to limited equipment and inadequate infrastructure. Doctor-to-ultrasound was the longest interval, reflecting constrained machine availability despite sufficient staffing. The study further concluded a need for system-level improvements such as expanding diagnostic capacity, redesigning pharmacy processes and use of task-shifting to enhance patient flow.

Although this study focused on data from a single department, it demonstrates the importance of time-motion analysis as an effective tool for identifying workflow bottlenecks and addressing limitations in workflow. These findings support the use of time-motion analyses across every department as an important aspect of enhancing workflow. It is recommended for future studies to analyze data across multiple departments and a broader time duration.

### Implications:

The findings of this study underscore the crucial role that digital innovations, particularly Electronic Health Records (EHRs) and Hospital Management Information Systems (HMIS) play in addressing bottlenecks in patient flow. Optimized HMIS integration can streamline registration, welfare categorization, and visit documentation, thereby reducing administrative delays and ensuring continuity of care. Regular time-motion analyses should be institutionalized for routine identification of bottlenecks and introducing relevant efficiency interventions. Embedding the time-motion analytics into the HMIS platforms can allow real-time monitoring of service utilization and waiting times, enabling proactive interventions. Such an integration will not only improve workflow efficiency but also enhance accountability, reduce medical errors, and strengthen overall quality of care. Efficient use of HMIS can help optimize scarce resources, improve supply chain management (e.g. pharmacy stock-outs) and reduce patient dropouts from care pathways. Thus, the integration of time-motion insights with digital health infrastructure represents a vital step towards building patient-centered, sustainable systems of healthcare delivery.

At a policy level, introducing pre-booking systems supported by the use of artificial intelligence and digital chat bots can make patient load predictable and prioritize high-need cases. The integration of HMIS and Electronic Medical Records systems at a broader level would enable inter-departmental coordination, and encourage data-driven decision making. Digital scheduling and appointment zoning based on case types can be introduced based on this data to balance daily workload and prevent crowding. Data-driven decisions for staffing adjustments and task redistribution can be made to relieve physicians from non-clinical duties, thereby improving quality of care.

### Author contribution:

**HKS:** study design, questionnaire design, data interpretation, and provided feedback through critical manuscript review.

**OT:** data collection and analysis.

**AM:** Literature search, data collection.

All authors have read the final version and are responsible and accountable for the accuracy and integrity of the work.

## References

[1] Taylor FW (1911). The principles of scientific management.

[2] Gilbreth FB, Gilbreth LM (1911). Motion study: A method for increasing the efficiency of the workman.

[3] Lopetegui M, Yen PY, Lai A, Jeffries J, Embi P, Payne P (2014). Time motion studies in healthcare: What are we talking about?. J Biomed Inform.

[4] Wang J, Lv H, Chen M, Liu C, Ren W, Jiang H (2025). A systematic review of lean implementation in hospitals: Impact on efficiency, quality, cost, and satisfaction. Int J Health Policy Manag.

[5] Ali H, Daud S (2025). A descriptive cross-sectional study on patient satisfaction with healthcare services provided in the outpatient department of a tertiary care hospital, Lahore. Life Sci.

[6] Chowdhury M, Maji S, Patra M, Chattopadhaya A (2024). A time-motion study and patient satisfaction in the fever clinic of a government medical college and hospital in India. Asian J Med Sci.

[7] Naaz F, Mohammed I (2019). A time motion study to evaluate the average waiting time in OPD with reference to patient satisfaction in a state-level AYUSH hospital (India). Med J Islam World Acad Sci.

[8] Manna NK, Samsuzzaman, Das S (2014). A time motion study in the OPD clinic of a rural hospital of West Bengal. IOSR J Dent Med Sci.

[9] Rizkiawan A, Pramesti AP, Pratiwi A (2019). A time-motion study description of nursing staff in medical unit of a hospital in Indonesia. KnE Life Sci.

[10] Majeed I, Faisal F, Shoukat M (2024). Optimizing patient flow in hospitals: Insights from machine learning algorithms. Pak J Med Res.

[11] Sujra MY, Hammad M, Ashraf R, Haider MI (2026). Overcoming obstacles and advancing the electronic health record system in Pakistan. J Bahria Univ Med Dent Coll.

[12] Ahmed RM, Qamar S (2024). Patient flow analysis and bottlenecks in tertiary care OPDs: A cross-sectional study from Karachi. Pak J Public Health.

[13] Zill EH, Huma S, Sania A, Sohail MM (2025). Between trust and tension: Psychosocial impacts of AI integration on health professionals'wellbeing. Int J Psychiatry Med.

[14] Haroon MZ, Thaver IH (2019). Patient flow and waiting time in emergency department of tertiary health care hospitals of Khyber Pakhtunkhwa. J Mech Cont Math Sci.

[15] Government of Pakistan (2025). Pakistan Economic Survey 2024-25: Health and nutrition.

[16] Molla S, Moges G, Toleha HN, Bayked EM, Workneh BD (2025). Patient satisfaction with pharmacy services and associated factors in Ethiopia: A systematic review and meta-analysis. BMC Health Serv Res.

[17] Batbaatar E, Dorjdagva J, Luvsannyam A, Savino MM, Amenta P (2017). Determinants of patient satisfaction: A systematic review. Perspect Public Health.

[18] Ullah S, Issa H (2024). The impact of physical surroundings on perception of service quality and patient satisfaction in public hospitals of Lahore. Pak J Int Aff.

[19] Tahir MN, Ch NA, Farooq MW, Mubin G, Kataria JR (2024). Situation analysis of the quality of primary health care services in Pakistan. East Mediterr Health J.

[20] Kanwel S, Ma Z, Li M, Hussain A, Erum N, Ahmad S (2024). The influence of hospital services on patient satisfaction in OPDs: Evidence from the transition to a digital system in South Punjab, Pakistan. Health Res Policy Syst.

